# Emerging Personality Pathology in Adolescents With Autism Spectrum Disorder: Clinical Correlates and Assessment Implications

**DOI:** 10.1177/13623613261476742

**Published:** 2026-09-06

**Authors:** Giacomo Distefano, Stefano Berloffa, Gianluca Sesso, Elena Valente, Antonio Narzisi, Guido Sorella, Chiara D’Ambrosio, Gabriele Masi, Annarita Milone

**Affiliations:** 1 IRCCS Stella Maris Foundation, Pisa, Italy; 2 University of Pisa, Italy; 3 Department of Mental Health and Pathological Addiction, AUSL Bologna, Italy; 4 IMT School for Advanced Studies, Lucca, Italy

**Keywords:** personality disorders, autism spectrum disorders, neurodevelopmental trajectories, child and adolescent psychiatry, female phenotypes of ASD

## Abstract

The link between neurodevelopmental and psychiatric conditions is increasingly recognized in developmental psychiatry, yet the relationship between Autism Spectrum Disorder (ASD) and Personality Disorders (PDs) remains underexplored in adolescence. Dysfunctional personality traits (DPT) may emerge in individuals with ASD, especially when diagnosis is delayed and comorbidities occur. This study assessed 79 adolescents (mean age = 15.15 ± 1.50; 45.6% females) with ASD without intellectual disability using a multimodal protocol (Autism Diagnostic Observation Schedule–Second Edition [ADOS-2], Autism Diagnostic Interview-Revised [ADI-R], Wechsler Intelligence Scale for Children–Fourth Edition [WISC-IV]/Wechsler Adult Intelligence Scale–Fourth Edition [WAIS-IV], Structured Clinical Interview for DSM-5 Personality Disorders [SCID-5-PD], Kiddie Schedule for Affective Disorders and Schizophrenia–Present and Lifetime Version [K-SADS-PL], standardized questionnaires). Analyses examined associations between DPT, psychiatric comorbidities, clinical severity, and functioning. DPTs were identified in 64.5% of participants, more frequently in females (75.0%) than males (48.9%). A prior ASD diagnosis was missing in 62%, particularly among females (odds ratio [OR] = 2.86, *p* = .030). Cluster A traits were most common (38.0%), notably Schizoid (22.8%), followed by Cluster B (24.1%), with Borderline traits markedly higher in females (22.2% vs. 2.3%, *p* = .006) and Cluster C (20.3%). DPTs were associated with lower functioning (Children’s Global Assessment Scale [CGAS], *p* = .037) and higher clinical severity (Clinical Global Impression [CGI], *p* < .019), also after False Discovery Rate (FDR) correction. Findings indicate that DPTs are highly prevalent yet often unrecognized in adolescents with ASD and are associated with greater comorbidity and poorer psychosocial outcomes. Early, developmentally informed assessment is crucial to identify risks and guide tailored interventions.

**Lay Abstract:**

Autism Spectrum Disorder (ASD) is a neurodevelopmental condition that affects social communication, behavior, and sensory processing. Some autistic adolescents may also show emerging maladaptive personality traits or early features of personality pathology. However, the relationship between ASD and personality disorders has been studied far less than other mental health conditions, especially during adolescence, a key developmental stage. This study included 79 adolescents with ASD without intellectual disability, with an average age of 15 years. Nearly half of the participants were female. Each adolescent completed a comprehensive clinical assessment, including standardized tools for autism diagnosis, psychiatric diagnostic interviews, cognitive testing, and questionnaires on emotional and behavioral functioning. We found that almost two-thirds of participants showed dysfunctional personality traits (DPTs). These traits were more common in females than males. In addition, many adolescents had not received an autism diagnosis before entering the study, and missed or delayed diagnosis was especially frequent among females. This is consistent with growing evidence that autism in girls may be less easily recognized, particularly when symptoms are masked or present differently from traditional expectations. The most common personality patterns involved social withdrawal, emotional detachment, and limited interest in close relationships. Other adolescents showed traits linked to emotional dysregulation, impulsivity, anxiety, perfectionism, or a strong need for control. Adolescents with DPTs also showed greater overall clinical severity and lower psychosocial functioning in everyday life, including difficulties with peers, family relationships, and school adjustment. These findings suggest that emerging personality pathology is common but often overlooked in autistic adolescents. If autism is identified late, emotional and interpersonal difficulties may become more complex over time. Clinicians should assess autistic traits, co-occurring psychiatric symptoms, and maladaptive personality features together, using a developmentally informed approach. Earlier and more accurate recognition may support personalized interventions, reduce distress, and improve outcomes during the transition to adulthood.

## Introduction

In recent years, the relationship between neurodevelopmental disorders and psychiatric disorders has garnered increasing attention in developmental psychiatry (Cristino et al., 2014; Morris-Rosendahl & Crocq, 2020; Thapar & Riglin, 2020). In this context, the link between Autism Spectrum Disorder (ASD) and Personality Disorders (PD) represents a relevant area of study because of its clinical implications (Rinaldi et al., 2021; Vuijk et al., 2018).

ASD is a neurodevelopmental disorder characterized by persistent deficits in social communication and restricted and repetitive patterns of behavior, interests, or activities (American Psychiatric Association, 2022). Its manifestations vary widely in severity, from significant daily impairment to relatively high-functioning ability, with normal or above-normal intelligence. The former classification into low- or high-functioning autism, despite being still extensively used in clinical practice, has been replaced in the *Diagnostic and Statistical Manual of Mental Disorders* (5th ed.; DSM-5; American Psychiatric Association, 2013) by a three-level severity system based on required support and individual autonomy (American Psychiatric Association, 2022). Level 1 includes the previous definitions of high-functioning ASD (HF-ASD) and Asperger’s syndrome. These subjects usually present milder forms of ASD but with a more insidious onset, often underestimated and difficult to diagnose, with significant developmental risks (Fucà et al., 2023; Rinaldi et al., 2021). Autistic people frequently show a high rate of psychiatric comorbidities, with a substantial impact on their quality of life. A recent estimate reports that 70% of individuals with ASD have a psychiatric comorbidity, and 40% may present more than one (American Psychiatric Association, 2022; Fusar-Poli et al., 2020; Lai et al., 2019). These rates may be even higher in individuals with ASD Level 1 (Fucà et al., 2023; Ooi et al., 2011). Understanding the prevalence, nature, and severity of these comorbidities is essential for developing effective interventions and support strategies. One of the comorbidities with a higher impact on daily functioning and clinical severity, but also less explored, in this population is PDs (Lai & Baron-Cohen, 2015).

PD is defined as:

an enduring pattern of inner experience and behavior that deviates markedly from the norms and expectations of the individual’s culture, is pervasive and inflexible, has an onset in adolescence or early adulthood, is stable over time, and leads to distress or impairment. (American Psychiatric Association, 2022)

The genesis of PD is complex and involves interactions among genetic, temperamental, and environmental factors, with a marked influence of early life experiences (Cloninger & Svrakic, 2008; Dixon-Gordon et al., 2015). Neurodevelopmental disorders, starting from the first years of life and stable over time, may be included among the “early life experiences.” Although no strict age criteria exist (except for antisocial PD, diagnosable only after age 18), PDs are rarely diagnosed in youth. While traditionally considered an adult-onset disease (Solmi et al., 2022), emerging evidence shows that dysfunctional personality traits (DPTs) in adolescence strongly predict adult PD (Benzi et al., 2023; De Clercq & De Fruyt, 2012; Sharp & Wall, 2018). PD are increasingly recognized in adolescence, with prevalence estimates of 10%–15% (Feenstra et al., 2011; Johnson et al., 2005), and research supports their diagnostic validity and temporal stability over time (Chanen & Kaess, 2012; Cohen et al., 2005; Sharp & Fonagy, 2015). For these reasons, in recent years, attention has focused on early recognition of DPT during childhood and adolescence. Several authors advocate the importance of analyzing PD from a developmental perspective, that is, taking into account the trajectory that led to a PD (Chanen & Kaess, 2012; De Fruyt & De Clercq, 2014). According to this theory, neurodevelopmental disorders can affect personality structuring and increase the risk of developing PD due to difficulties in emotional regulation, inconsistency in the perception of self and others, and insecurity in interpersonal relationships (Fountain et al., 2023; Lugnegård et al., 2012). Within this framework, the relationship between ASD and PD remains complex and underexplored. In individuals with ASD without intellectual disability (ID), delayed diagnosis and impaired social development may disrupt typical personality maturation, leading to rigid interpersonal patterns and a sense of social exclusion (Fountain et al., 2012; Fusar-Poli et al., 2020). Beyond neurodevelopmental characteristics, personality development in autistic adolescents is also influenced by environmental and relational experiences, such as social exclusion and bullying (Abregú-Crespo et al., 2024; Hoover, 2015), as well as the cumulative burden of sensory distress (Corbett et al., 2016) and camouflaging required to live in a predominantly non-autistic world, which may contribute to stress and shape developmental trajectories.

Recent systematic literature reviews report that 48%–62% of adults with ASD meet criteria for at least one PD (Rinaldi et al., 2021; Vuijk et al., 2018) or that individuals with PD may present significantly higher ASD traits compared to non-clinical samples (Gillett et al., 2023). Research highlights significant associations between ASD and Cluster A and C PDs, especially obsessive-compulsive, schizoid, schizotypal, and avoidant PDs (Anckarsäter et al., 2006; Cook et al., 2020; Hofvander et al., 2009; Lugnegård et al., 2012). The overlap between ASD and PD, given the egosyntonic characteristics and common traits such as rigidity, perfectionism, and low social functioning, complicates the differential diagnosis, highlighting the need for a specific assessment to distinguish between these two conditions (Bach & Vestergaard, 2023; Lai & Baron-Cohen, 2015).

Most research investigating the relationship between ASD and PD focuses on adult samples, leading to the absence of developmental-focused studies. This gap represents a critical point in understanding personality development in this population since many of its characteristics start to manifest at a young age. In addition, the complexity of diagnostic assessment of ASD in the adult population, which requires a developmental history that is often difficult to reconstruct, introduces further obstacles in differentiating between ASD and PD.

Based on this theoretical framework, our study aims to investigate the presence of DPT in a clinical population of adolescents with Level 1 ASD, applying rigorous assessments for neuropsychological and psychopathological variables, with particular attention to the evaluation of ASD symptoms and neurodevelopmental history. A key goal is to clarify the overlap and distinction between ASD and PD, under the hypothesis that certain neurodevelopmental conditions may increase vulnerability to, or shape the later emergence of, personality pathology.

The second objective was to explore influencing risk and co-occurring factors, such as psychiatric comorbidities, that may predispose individuals with ASD to develop PDs. Particular attention will be given to gender differences and the utility of standardized diagnostic tools.

## Methods

### Sample

Seventy-nine patients aged between 13 and 18 with ASD and normal cognitive functioning, referred between April 2022 and December 2024 to our third-level Department of Child and Adolescent Psychiatry and Psychopharmacology (IRCCS Fondazione Stella Maris – Calambrone [Pisa] Italy) for psychiatric symptoms (e.g., mood and anxiety disorders) associated with significant socio-relational impairment requiring diagnostic clarification, were included in the study. The autism diagnosis was either previously established or confirmed following our comprehensive multimodal assessment. The total sample included 36 females (45.6%) and 43 males (54.4%). The mean age of the participants was 15.15 years (*SD* = ±1.50), ranging from 13 to 18 years.

Exclusion criteria were as follows: age < 13 or > 18 years old, failure to confirm ASD diagnosis through standardized assessments, ID or borderline intellectual functioning (intelligence quotient [IQ] < 70 or General Ability Index [GAI] < 84), neurological disorders or significant hearing/visual deficits, and active psychotic symptoms.

Informed consent was obtained from all participants and their legal representatives. The study adhered to the tenets of the Declaration of Helsinki and was approved by the Ethics Committee.

The initial sample consisted of 84 participants. Following enrollment, five participants were excluded: two did not meet *Diagnostic and Statistical Manual of Mental Disorders* (5th ed., text rev.; DSM-5-TR; American Psychiatric Association, 2022) criteria for ASD after comprehensive assessment, one did not meet the study’s cognitive inclusion criteria, and two did not meet the age inclusion criteria (aged 11 and 12 years). These participants were excluded a priori from all statistical analyses. The final sample, therefore, consisted of 79 participants. The number of individuals who declined participation was not systematically recorded; however, this number was expected to be minimal, as the study protocol closely reflected routine clinical assessment procedures in our service.

### Measures

#### ASD Assessment

All participants, regardless of the presence of a prior clinical diagnosis of autism, underwent a comprehensive multimodal assessment. A total of 77 patients were administered the Autism Diagnostic Observation Schedule–Second Edition (ADOS-2; Lord et al., 2012) by an experienced clinical psychologist to support ASD diagnosis and obtain comparison scores (CSs). ADOS-2 was not administered to two patients due to logistical issues or severe clinical conditions. In 51 cases, where diagnostic uncertainty remained after clinical evaluation and ADOS-2, the Autism Diagnostic Interview-Revised (ADI-R; Lord et al., 1994) was conducted with parents to further clarify the diagnosis.

Autism diagnosis was established through an integrated clinical evaluation based on DSM-5-TR criteria, in which standardized instruments were used to support, but not replace, clinical judgment. Patients who did not meet DSM-5-TR criteria for ASD following clinical evaluation and standardized assessment were excluded.

#### Cognitive Assessment

A standardized cognitive assessment was administered to all patients using *WISC-IV* (*Wechsler Intelligence Scale for Children–Fourth Edition*) (Wechsler, 2012b) if under 16 years old or *WAIS-IV* (*Wechsler Adult Intelligence Scale–Fourth Edition*) (Wechsler, 2012a) if above 16 years old. All patients who did not have a total IQ above 85 or a total IQ between 70 and 84 with GAI above 85 were excluded from the present study.

#### Dysfunctional Traits of Personality Assessment

For this study, we adopted the classical categorical model for PDs as defined in the DSM-5-TR. Although dimensional approaches are increasingly recommended for younger populations, the categorical model remains widely used in adolescent clinical research due to its extensive validation, clear diagnostic structure, and comparability with prior studies.

All participants were first administered the SCID-5-SPQ (Structured Clinical Interview for DSM-5–Screening Personality Questionnaire), followed by the SCID-5-PD (Structured Clinical Interview for DSM-5 Personality Disorders) (First et al., 2017) to explore the presence of DPT according to the DSM-5-TR categorical framework. The interviews were conducted by a licensed clinical psychologist with expertise in PDs and prior experience with adolescent populations. Although the SCID-5-PD is formally standardized for adults, its use was considered appropriate given the participants’ age range and the instrument’s high clinical reliability and communicative clarity.

Standard SCID-5-PD diagnostic thresholds were applied during the interview. However, for developmental and ethical reasons, threshold-level findings were reported as DPTs rather than formal PD diagnoses. In fact, in line with ethical and developmental considerations, we did not aim to establish categorical PD diagnoses; rather, we focused on identifying and describing patterns of DPTs. Minor linguistic and contextual adaptations were applied to enhance developmental appropriateness, and all results were interpreted with caution, taking into account the ongoing maturation of personality during adolescence (see Supplementary Materials – SCID-5-PD Adaptations for Adolescent Assessment). Both the SCID-II-PD and the SCID-5-PD have previously been applied in adolescent samples to assess personality pathology (Gander et al., 2024; Jørgensen et al., 2024; Somma et al., 2019).

#### Psychopathological Assessment

All patients and parents of patients were administered the *K-SADS-PL* (*Kiddie Schedule for Affective Disorders and Schizophrenia–Present and Lifetime Version*) (Kaufman et al., 2004) semi-structured interview by a child psychiatrist with clinical expertise in developmental psychiatry to assess the presence of psychopathological disorders diagnosable by DSM-5-TR criteria.

After the comprehensive clinical assessment, two clinicians with expertise in developmental psychiatry completed the *Children’s Global Assessment Scale* (*CGAS*) (Shaffer et al., 1983) and *Clinical Global Impression* (*CGI*) (Guy, 1976) scales to assess the patient’s functioning and clinical severity. The clinicians were different from the examiner who conducted the personality assessment, and these raters were not informed of the SCID-5-PD results at the time of rating.

#### Clinical Questionnaires

All patients completed self-report questionnaires to explore key psychopathological dimensions, although clinical severity led to incomplete data in some cases. Self-report tools included the *YSR 11/18* (Achenbach, 2001) for internalizing/externalizing symptoms, *RIPoSt-Y* (Sesso et al., 2021) for emotional dysregulation, *MASC 2™-SR* (March, 2013) for anxiety, and the *ICU* (Essau et al., 2006) for callous-unemotional traits.

Parents completed parallel measures such as *CBCL 6/18* (Achenbach, 2001), *CPRS-R:S* (Conners et al., 1998), and *SCQ* (Rutter, 2003) for behavioral and developmental screening. A subset also completed the *JTCI – Caregiver Version* (Luby et al., 1999), although related analyses are still ongoing and results are not reported here.

### Statistical Analysis

All analyses were performed using IBM SPSS Statistics^®^ (version 30.0). First, descriptive analyses of key sociodemographic and clinical variables were conducted to characterize the sample. Data distribution was then evaluated with the Kolmogorov–Smirnov and Shapiro–Wilk tests to determine whether to apply parametric or non-parametric methods. For comparisons between two groups, the independent-samples *t*-test was employed when the data were normally distributed and displayed homogeneous variances (Levene’s test). Otherwise, when the assumption of homogeneity of variance was violated or the distribution was non-normal, the Mann–Whitney test was utilized. A False Discovery Rate (FDR) correction was applied using the Benjamini–Hochberg procedure following the *t*-test or Mann–Whitney analyses to control for multiple comparisons.

Pearson’s chi-square test was performed to assess associations between categorical variables. Whenever the expected frequencies in any cell were below 5, Fisher’s exact test was used where appropriate.

To examine group differences, analysis of covariance (ANCOVA) models were performed for the main clinical outcomes (CGAS, CGI, and ADOS-2 CS), controlling for potential confounders. Each ANCOVA included Cluster membership (A, B, C, combined, no DPT) as the fixed factor, and age, sex, and total IQ as covariates. Homogeneity of variances (Levene’s test) and normality of residuals (Shapiro–Wilk test) were verified before running the analyses, and the homogeneity of regression slopes assumption was met in all models. Partial eta-squared ( $\eta_{p}^{2}$ ) was used as an index of effect size, with values of .01, .06, and .14 representing small, medium, and large effects, respectively. Post hoc pairwise comparisons were conducted using Tukey’s honestly significant difference (HSD) with 95% confidence intervals (CIs) and Cohen’s *d* effect sizes.

The significance level was set at *p* < .05. Where applicable, effect sizes were calculated for parametric tests (e.g., Cohen’s *d* for *t*-tests), and rank-biserial correlations were employed for non-parametric tests to evaluate the magnitude of differences.

## Results

### Clinical Characteristics of the Sample

The CGAS was used to assess the global level of functioning, with the sample showing a mean score of 45.77 (*SD* = ± 8.85) (Table 1). In parallel, clinical severity measured through the CGI showed a mean of 4.61 (*SD* = ±1.04). The distribution of CGI scores confirmed that the most represented group in the sample was those with moderate severity (score of 5, 40.5%), followed by those with “medium” severity (score of 4, 27.8%). A significant part of the sample had 6 (12.7%) and 7 (3.8%) scores, indicating a more severe clinical picture. To investigate autistic symptomatology, the ADOS-2 was administered. The CS was available for 77 subjects (two data missing), with a mean score of moderate autistic symptoms (*M* = 5.43, *SD* ± 1.53). ADOS-2 severity levels are reported in Table 1. Regarding the cognitive profile, the mean scores of total IQ (*M* = 110.52; *SD* = ± 18.58), VCI (Index of Verbal Comprehension; *M* = 116.65; *SD* = ± 17.84), and PRI (Perceptual Reasoning Index; *M* = 112.65; *SD* = ±15.66) are above the expected mean for age. In contrast, there is a decline in WMI (Working Memory Index; *M* = 95.94, *SD* = ± 20.31) and PSI (Processing Speed Index; *M* = 91.56, *SD* = ±17.57) indices.

**Table 1.** Demographic and Clinical Characteristics of the Sample (*N* = 79).

Characteristics	Value
Age (years)	*M* = 15.15 (*SD* = 1.50), range = 13–18
Sex, *n* (%)	Female: 36 (45.6%), Male: 43 (54.4%)
Children’s Global Assessment Scale (CGAS)	*M* = 45.77 (*SD* = 8.85)
Clinical Global Impression (CGI)	*M* = 4.61 (*SD* = 1.04)
Autism Diagnostic Observation Schedule (ADOS-2) (*n* = 77)	*M* = 5.43 (*SD* = 1.53)
ADOS Severity Levels, *n* (%):	Mild: 27.8% Moderate: 59.5% Severe: 10.1%
Full-Scale IQ	*M* = 110.52 (*SD* = 18.58)
Verbal Comprehension Index (VCI)	*M* = 116.65 (*SD* = 17.84)
Perceptual Reasoning Index (PRI)	*M* = 112.65 (*SD* = 15.66)
Working Memory Index (WMI)	*M* = 95.94 (*SD* = 20.31)
Processing Speed Index (PSI)	*M* = 91.56 (*SD* = 17.57)

*Note.* The ADOS-2 comparison score was not available for two participants.

### Missed Diagnosis of ASD and Distribution by Sex

Of the 79 patients enrolled in the present study, only 30 already had a prior diagnosis of ASD at the time of our evaluation, while 49 patients (62%) had no diagnosis at the time of evaluation and had accessed child psychiatric services for other psychiatric issues (e.g., mood disorders, anxiety disorders, etc.). Analyzing the distribution of missed ASD diagnoses within the two sexes shows that ASD diagnosis before assessment was present in only 25.0% of the female sample compared with 48.8% of the males. Overall, there was a significant difference in the distribution of the two variables measured with chi-square, with a higher prevalence of females observed among patients with no diagnosis of ASD at the time of our assessment (Pearson’s χ^2^ = 4.727; *p* = .030; odds ratio [OR] = 2.86).

### Psychiatric Comorbidities

Patients were recruited from a clinical sample of adolescents accessing a third-level center for other psychiatric conditions, so individuals showed complex clinical pictures with high comorbidity rates. Mood disorders (79.7%) and anxiety disorders (84.8%) were the most represented psychiatric comorbidities. Confirming the clinical severity of the sample is a high rate of suicidal behaviors (27.9%; specifically, suicidal ideation 22.8% and suicidal attempt 5.1%) and self-injurious behaviors (25.3%). The prevalence of other neurodevelopmental disorders in comorbidity is also significant, at 67.1%, encompassing Attention-Deficit/Hyperactivity Disorder (ADHD; 58.2%), Specific Learning Disorders (27.8%), and Tic/Tourette’s syndrome (10.1%). The distribution of all psychiatric comorbidities within the sample is shown in Table 1 of the Supplementary Material.

### DPTs

The study identified a high prevalence of DPT within the sample, with an overall rate of 64.5%. This prevalence was notably higher among females, reaching 75%, compared to 48.9% in males (Table 2). Statistical analysis revealed a trend toward significance in the association between sex and the overall presence of DPT (*p* = .076). Regarding the distribution across diagnostic clusters, Cluster A traits were the most represented, accounting for 38.0% of the sample. Within this cluster, Schizoid traits were the most frequent (22.8%), followed by Paranoid (16.5%) and Schizotypal (8.9%) traits. Schizoid traits were more prevalent in males (27.9%) than in females (16.7%).

**Table 2.** Dysfunctional Personality Traits Distribution.

Personality disorder	Total (*n* = 79)		Female (*n* = 36)		Male (*n* = 43)	
	*n*	%	*n*	%	*n*	%
Any Personality Disorder	51	64.5%	27	75.0%	24	48.9%
Cluster A	30	38.0%	14	38.9%	16	37.2%
Paranoid DP	13	16.5%	8	22.2%	5	11.6%
Schizotypal DP	7	8.9%	4	11.1%	3	6.9%
Schizoid DP	18	22.8%	6	16.7%	12	27.9%
Cluster B	19	24.1%	13	36.1%	6	13.9%
Histrionic DP	3	3.8%	2	5.6%	1	2.3%
Narcissistic DP	12	15.2%	8	22.2%	4	9.3%
Borderline DP	9	11.4%	8	22.2%	1	2.3%
Antisocial DP	0	0.0%	0	0.0%	0	0.0%
Cluster C	16	20.3%	9	25.0%	7	16.2%
Avoidant DP	13	16.5%	7	19.4%	6	13.9%
Dependent DP	0	0.0%	0	0.0%	0	0.0%
Obsessive-compulsive DP	5	6.3%	4	11.1%	1	2.3%

*Note.* Some individuals exhibited dysfunctional personality traits attributable to multiple clusters.

Cluster B was present in 24.1% of participants. Borderline Personality traits stood out within this group, with an overall prevalence of 11.4%, and a marked sex difference: 22.2% in females versus 2.3% in males. A significant association was found between female sex and Cluster B traits (χ^2^ = 5.27; *p* = .022), with an OR of 3.49. Notably, a highly significant association emerged between sex and Borderline Personality traits (χ^2^ = 7.685; OR = 12; *p* = .006), confirming a markedly higher prevalence in females. Narcissistic Personality traits were observed in 15.2% of the sample, and Histrionic Personality traits in 3.8%. No cases of Antisocial Personality traits were detected, due to the impossibility of diagnosing it before 18 years old.

Cluster C accounted for 20.3% of the cases. Avoidant Personality traits emerged as the most common subtype (16.5%), whereas Dependent Personality traits were absent (0%). Obsessive-Compulsive Personality traits were present in 6.3% of the sample. Associations between specific DPT or Clusters and Psychiatric Comorbidities are available in Table 2 of the Supplementary Material.

### Comparison Between Subjects With DPT Versus Subjects Without DPT

To explore differences between individuals with and without DPT, we conducted a series of analyses using independent-samples *t*-tests and Mann–Whitney *U* tests, where appropriate. We then applied the FDR correction using the Benjamini–Hochberg method. The groups were compared across a range of clinical, cognitive, and psychopathological variables. The comparison between individuals with and without DPT revealed clear and clinically meaningful differences across several domains. There were no significant group differences in terms of age, cognitive functioning, or autism symptom severity (see Table 3 of the Supplementary Material). However, participants with DPT showed a marked reduction in global functioning, as indicated by significantly lower CGAS scores (*M* = 43.65 vs. 49.64; corrected *p* = .037), and a corresponding increase in clinical severity, as reflected in higher CGI scores (*M* = 4.92 vs. 4.04; corrected *p* = .019) (see Table 4 of the Supplementary Material). Differences between groups were also evident in emotional and behavioral symptoms reported both by caregivers and the adolescents themselves. On the Child Behavior Checklist (CBCL), those with DPT showed significantly elevated internalizing symptom scores (corrected *p* = .049) and more pronounced affective problems (corrected *p* = .037) (see Table 4 of the Supplementary Material).

The Youth Self Report (YSR) further confirmed this profile. Youth with DPT reported significantly higher internalizing symptoms (corrected *p* = .049), as well as more severe withdrawn/depressed symptoms (corrected *p* = .019), rule-breaking behavior (corrected *p* = .019), and affective problems (corrected *p* = .037) (see Table 4 of the Supplementary Material). Further differentiation emerged in measures of affective and personality traits. Participants with DP displayed greater affective instability, as measured by the RIPoSt-Y (corrected *p* = .082, uncorrected *p* = .025), and higher levels of callousness on the ICU (corrected *p* = .066, uncorrected *p* = .012). Although these did not meet the stricter threshold for statistical significance after correction, they suggest meaningful trends that warrant further investigation (see Table 5 of the Supplementary Material).

### Comparison Between Clusters

To explore differences among personality clusters (A, B, C, and combined clusters) and the absence of DPT, an ANCOVA was conducted on a range of clinical variables. The analysis revealed significant group effects on several measures, while others did not show statistically meaningful differences. Specifically, no significant differences emerged between groups in terms of age or cognitive indices, including total IQ, VCI, PRI, WMI, and PSI, indicating comparable cognitive profiles across the sample. Significant group differences were also found for the CGAS after controlling for age, sex, and IQ. The overall effect was statistically significant, *F*(4,69) = 2.85, *p* = .030, $\eta_{p}^{2}$ = 0.14, with post hoc analyses showing that individuals in the No DPT group had significantly higher functioning compared to those in Cluster B (*p* = .030, Cohen’s *d* = 1.10). On average, participants without PDs reported the highest adjusted CGAS scores (*M* = 49.3, 95% CI = [45.9–52.7]), suggesting better overall functioning, while those in Cluster B had the lowest (*M* = 40.0, 95% CI = [34.8–45.1]).

A similar pattern was observed for clinical severity, as measured by the CGI. The ANCOVA indicated a significant main effect of cluster, *F*(4,69) = 3.10, *p* = .021, $\eta_{p}^{2}$ = 0.15, with post hoc comparisons revealing that the No DPT group differed significantly from the Combined Cluster (*p* = .034) and nearly from Cluster B (*p* = .064). Individuals without PDs showed lower adjusted CGI scores (*M* = 4.11, 95% CI = [3.71–4.50]), whereas those in Cluster B (*M* = 5.08, 95% CI = [4.48–5.67]) and the Combined Cluster (*M* = 5.15, 95% CI = [4.59–5.71]) exhibited greater clinical severity.

Finally, significant differences between groups were also found in the ADOS-2 CSs. The ANCOVA was significant, *F*(4,67) = 2.79, *p* = .033, $\eta_{p}^{2}$ = 0.14, with post hoc analyses indicating that Cluster A participants had higher adjusted ADOS scores compared to those in the Combined Cluster (*p* = .025, Cohen’s *d* = 1.21). Specifically, Cluster A showed the highest mean ADOS scores (*M* = 6.12, 95% CI = [5.36–6.88]), while the Combined Cluster had the lowest (*M* = 4.32, 95% CI = [3.46–5.17]).

## Discussion

To our knowledge, this is the first study to investigate the presence of DPTs in a sample of adolescents with ASD (level 1 for DSM-5-TR criteria) by conducting a complete and structured assessment regarding psychopathological, cognitive, and personological features and associating a comprehensive reassessment of ASD with validated instruments (ADOS-2 and ADI-R). Some studies examined the prevalence of PDs in autistic adolescents, specifically concentrating on Cluster A due to symptomological continuity with schizoid PD (Barneveld et al., 2011; Cook et al., 2020), rather than evaluating PDs comprehensively. Overall, our findings confirm the results of studies on adult populations with ASD (Anckarsäter et al., 2006; Lugnegård et al., 2012), highlighting, on the contrary, the importance of early diagnosis to intercept dysfunctional developmental trajectories in this population (Fountain et al., 2012).

Our data provide a complex and heterogeneous picture of personality profiles in adolescents with ASD without ID. First, the fact that 62% of the participants had no prior diagnosis of ASD at the time of our evaluation confirms the challenge of identifying ASD features in a population with normal cognitive functioning (Lai & Baron-Cohen, 2015; Rinaldi et al., 2021). This diagnostic delay, combined with years of compensatory strategies to hide autistic symptoms, leads to a high cost in terms of stress and cognitive effort, negatively influencing the structuring and maturation of the personality (Kose et al., 2023; Narzisi & Stavropoulos, 2022). Second, the significant association between female sex and failure to recognize the disorder suggests the importance of identifying and describing the so-called “female phenotype” of ASD, which is characterized by specific clinical features, often accompanied by greater adaptation and masking strategies (“social camouflage”), leading to diagnostic delay (Allely, 2018; Hull et al., 2020).

Overall, a high rate of psychiatric comorbidities has emerged in our sample, particularly mood and anxiety disorders. These comorbidities, on one hand, can bring the patient to clinical attention, and, on the other hand, they can mask the diagnosis, orienting clinicians toward other psychopathological conditions (Fusar-Poli et al., 2020). Very significant data also emerge regarding the prevalence of suicidality (27.9%) and self-injurious behaviors (25.3%), reflecting the vulnerability of autistic individuals in adolescence, characterized by fragility in emotional regulation and difficulties in processing social relationships (Goldsmith & Kelley, 2018; Samson et al., 2015). In addition, the association between psychiatric disorders and PD is widely acknowledged; therefore, assessing psychiatric comorbidities is crucial to understand the genesis and progression of PD (Friborg et al., 2014; Khan et al., 2005).

The main finding of our study is the high prevalence (64.5%) of DPT in our sample, which increases to 75% in females, in line with other studies on clinical populations (Klinkby et al., 2024). Our findings align with systematic reviews that report the presence of a PD in 24%–60% of autistic adults (Rinaldi et al., 2021; Vuijk et al., 2018) and high rates of autistic traits in adults with PD (Gillett et al., 2023). Although the majority of studies focus on adults, some previous work suggests that the co-occurrence of ASD and DPT may appear as early as adolescence (Barneveld et al., 2011; Cook et al., 2020; Rosello et al., 2023). In terms of diagnostic terminology, we have opted for the term “dysfunctional personality traits” instead of the more definitive “personality disorders,” considering the young age of patients and their potential for personality maturation. Nevertheless, our data suggest that the overlap between ASD and PD is not a uniquely “late” phenomenon but may occur before reaching adulthood, and that it is therefore crucial to intercept these traits as early as possible (Fountain et al., 2012; Rinaldi et al., 2021). In this perspective, the term “comorbidity” may be questioned, as neurodevelopmental disorders affect the development of personality since the beginning, with specific “influences” determined by psychopathological dimensions such as impulsivity, delay aversion, emotional dysregulation, lack of empathy, and social disturbances, which may direct personality development in specific deviant trajectories. We use the term “comorbidity” to describe the final destination of these developmental pathways.

Most studies in adult populations indicate that PDs belonging to Clusters A and C are the most strongly associated with ASD (Anckarsäter et al., 2006; Gadelkarim et al., 2019; Lugnegård et al., 2012; Zhou et al., 2019). ASD and these PDs share some clinical aspects, for example, social-emotional detachment (in schizoid ASD), eccentricity (in schizotypal ASD), and preoccupation with details, order, and inflexibility (in obsessive-compulsive ASD) (Lai & Baron-Cohen, 2015). In our sample, Cluster A (38.0%) was the most frequently represented, consistent with the literature reporting (Anckarsäter et al., 2006; Barneveld et al., 2011). As expected, Schizoid Personality Disorder (22.8%) was found to be the most frequent, given the continuity in symptoms and presentation of these two disorders, characterized by isolation, emotional detachment, and interpersonal difficulties (Cook et al., 2020; Hrdlicka & Dudova, 2013). However, higher prevalence of paranoid (22.2% vs. 11.6%) or schizotypal (11.1% vs. 6.9%) traits emerged in females compared to males. One possible explanation is that females with ASD often engage in social camouflaging, leading to increased internal distress, hypervigilance, and social anxiety, manifesting as paranoid or schizotypal features (Hull et al., 2020; Lai et al., 2019). In addition, the increased likelihood of social trauma in autistic females (e.g., bullying, social rejection) may contribute to the development of paranoid or schizotypal coping mechanisms (Cook et al., 2020).

Cluster B (24.1%), on the contrary, correlates with mood disorders (particularly bipolar-cyclothymic spectrum), suicidality, and self-injurious behaviors, highlighting the complexity of managing these patients (Dell’Osso et al., 2018; Rydén et al., 2008). Borderline traits appear notably more frequent in females (22.2%) than males (2.3%), aligning with adult data on overlapping features between ASD and Borderline PD (Dudas et al., 2017; Fusar-Poli et al., 2020; May et al., 2021). Recent research highlights shared difficulties in mentalization and emotional regulation across ASD and borderline PD (Dudas et al., 2017; Vegni et al., 2021). Emotional dysregulation, characterized by mood instability and relational impairments, is central to Borderline Personality Disorder (BPD) development (Fitzpatrick et al., 2023). In autistic females, heightened limbic activity may contribute to increased emotional reactivity and atypical beliefs, potentially overlapping with BPD features (Calderoni et al., 2012; Carroll & Chiew, 2006).

As regards Narcissistic PD, individuals with ASD may develop narcissistic-like traits as a result of core features such as Theory of Mind deficits and impaired social reciprocity, which foster egocentric behavior and poor perspective-taking (Baron-Cohen, 1995; Broglia et al., 2024; Frith, 2001). Chronic social rejection and identity confusion can lead to compensatory self-focus and rigid self-image, resembling vulnerable narcissism (Hull et al., 2020; Ronningstam, 2005).

Finally, Cluster C (20.3%) correlates with anxiety disorders, confirming the tendency of adolescents with ASD to experience marked anxiety vulnerability that, when structured on a personological basis, takes on unchanging characteristics of avoidance, rigidity, or hypercontrol (M. C. Lai & Baron-Cohen, 2015). Avoidant PD, in particular, shows an association with social anxiety that appears clinically relevant and clarifies how “shy-inhibited” ASD, with reduced social skills, can easily lead to a personological pattern of avoidance (Barneveld et al., 2011).

Compared to those without DPT, individuals with DPT showed poorer global functioning (lower CGAS), higher clinical severity (higher CGI), and higher internalizing, affective, withdrawal, and depressive symptoms on both CBCL and YSR. These findings indicate that DPTs are associated with worse clinical outcomes and more complex management, in line with evidence from adult populations (Lugnegård et al., 2012; Rinaldi et al., 2021).

Personality cluster membership was significantly associated with both functional and clinical outcomes: Cluster B traits were linked to poorer psychosocial functioning and greater clinical severity, whereas Cluster A traits were associated with higher levels of autistic symptomatology. These associations remained significant after controlling for age, sex, and IQ. Overall, these results highlight the clinical relevance of personality dimensions in ASD, with Cluster B traits exacerbating functional impairment and Cluster A traits potentially reflecting core autistic features.

The interpretation of personality pathology in autistic adolescents should be approached with caution, as several features assessed by instruments such as the SCID-5-PD (e.g., social withdrawal, rigidity, emotional dysregulation) may overlap with core autism characteristics or represent adaptive responses to environmental stressors in this population. As previously highlighted (Lai & Baron-Cohen, 2015), some presentations (particularly within Cluster A and Cluster C) may reflect shared phenotypic expressions rather than distinct co-occurring conditions.

In this perspective, the construct of PD in neurodevelopmental populations appears inherently complex and may require reconsideration within a developmental and neurodiversity-informed framework. The observed overlap may therefore reflect, at least in part, the limits of current categorical diagnostic systems in disentangling autism-related features from personality-related constructs (e.g., McQuaid et al., 2024). These considerations support a shift toward more dimensional approaches to the assessment of personality functioning, which may be better suited to capturing the heterogeneity and developmental fluidity of these presentations. A dimensional perspective may permit a deeper characterization of individual profiles, reducing the risk of misinterpretation of autism-related features as distinct personality pathology and improving clinical formulation in this population.

Our findings highlight the clinical importance of a multimodal assessment in ASD, particularly during adolescence (Goldsmith & Kelley, 2018), through the systematic use of standardized assessment tools. Careful reconstruction of early developmental history is crucial to prevent misdiagnosis or diagnostic delay (Fusar-Poli et al., 2020). Standardized retrospective tools like the ADI-R should complement clinical observation. Moreover, early screening for personality traits is essential, as adolescence may reveal latent trajectories toward PDs.

While this study offers new insights into the meaning of “comorbidity” between ASD and PD during adolescence, several limitations must be considered. The sample size limits advanced subgroup and statistical analyses, particularly for less-represented PDs. Although refusal rates were expected to be low because the study procedures largely overlapped with routine clinical care, the number of eligible patients who declined participation was not systematically recorded. Therefore, a potential impact of non-participation on the generalizability of the findings cannot be entirely excluded. The highly specialized clinical setting introduces a sampling bias, as participants were referred for complex psychiatric presentations and diagnostic clarification, often in the context of previously unrecognized autism. This likely inflates the observed rates of co-occurring personality pathology and limits the generalizability of findings to clinically referred populations rather than the broader autistic population. The absence of a control group restricts normative comparisons. As regards sex distribution, a small subgroup of participants reported features consistent with gender dysphoria; however, gender identity was not systematically assessed and was therefore not included in the analyses, representing a limitation of the study. The symptomatic overlap with ADHD and other neurodevelopmental conditions complicates differential diagnosis, underscoring the need for standardized protocols and longitudinal studies to clarify developmental trajectories (May et al., 2021). Moreover, reliance on self-report measures in ASD may be affected by limited self-awareness and reflective capacity, associated with data loss due to lower patient compliance. Broader, multicenter case-control studies are needed, along with research focusing on shared neurobiological substrates of ASD and PD and transdiagnostic dimensions, such as empathy, mentalization, and emotional dysregulation, for better clinical translation.

## Conclusions

Pervasive developmental problems related to psychosocial functioning in ASD appear to alter personality maturation, resulting in impoverished interaction with the surrounding environment, feelings of exclusion, and experiences of social distress. The presence of DPT in youth is associated with lower psychosocial functioning, greater clinical severity, and increased emotional and behavioral difficulties. Longitudinal studies have shown that the majority of adolescents who meet criteria for PD experience marked impairments in social and academic functioning (Crawford et al., 2008; Gunderson et al., 2011), with a higher risk of contacts with the psychiatric emergency departments (Klinkby et al., 2024).

The emergence of a high rate of psychiatric comorbidity and the presence of DPT as early as adolescence suggest the urgency of a clinical approach that also pays attention to the genetic predisposition, neurobiological features, early history, and environmental exposure of each individual. In this sense, it is crucial to focus on understanding developmental trajectories from the earliest stages of development, as a late recognition of ASD or other neurodevelopmental disorders, as well as an underestimation of affective comorbidities, can significantly affect the prognosis and quality of life of the individual. Future research, involving larger samples and longitudinal designs from earlier ages, is expected to deepen the characteristics of this complex clinical interaction, yielding more precise insights for early diagnosis and the formulation of effective and individualized intervention strategies.

## Supplemental Material

sj-docx-1-aut-10.1177_13623613261476742 – Supplemental material for Emerging Personality Pathology in Adolescents With Autism Spectrum Disorder: Clinical Correlates and Assessment Implications Supplemental material, sj-docx-1-aut-10.1177_13623613261476742 for Emerging Personality Pathology in Adolescents With Autism Spectrum Disorder: Clinical Correlates and Assessment Implications by Giacomo Distefano, Stefano Berloffa, Gianluca Sesso, Elena Valente, Antonio Narzisi, Guido Sorella, Chiara D’Ambrosio, Gabriele Masi and Annarita Milone in Autism

## Data Availability

The data and code supporting the findings of this study are not publicly available due to ethical and privacy restrictions involving minor participants. De-identified data may be made available from the corresponding author upon reasonable request and approval by the Ethics Committee.
