# Supplementary material for: Emerging Personality Pathology in Adolescents With Autism Spectrum Disorder: Clinical Correlates and Assessment Implications
Source: Autism. 2026 Sep 6;30(11):2739–51. doi: 10.1177/13623613261476742 (PMC13687640; doi:10.1177/13623613261476742)
Supplement: Supplementary file 1 [file sj-docx-1-aut-10.1177_13623613261476742.docx]

**Supplementary Materials**

**Table 1 – Suppl**

*Frequency of psychiatric comorbidities in the sample*

| **Psychiatric comorbidity** | **Patients (n)** | **Patients (%)** |
| --- | --- | --- |
| **Other** **neurodevelopmental disorders** | 53 | 67.1 |
| ADHD | 46 | 58.2 |
| Specific learning disorder | 22 | 27.8 |
| Tic/Tourette's | 8 | 10.1 |
| **Mood disorders** | 63 | 79.7 |
| Bipolar spectrum | 42 | 53.2 |
| Depression | 21 | 26.6 |
| **Anxiety disorders** | 67 | 84.8 |
| Multiple anxiety | 64 | 81.0 |
| Social anxiety | 58 | 73.4 |
| Performance anxiety | 49 | 62.0 |
| Somatic symptoms | 36 | 45.6 |
| School phobia | 15 | 19.0 |
| Separation anxiety | 13 | 16.5 |
| **Impulse control disorders** | 35 | 44.3 |
| Emotional dysregulation | 34 | 43.0 |
| Oppositional-defiant disorder | 9 | 11.4 |
| Conduct disorder | 2 | 2.5 |
| **Suicidality** | 22 | 27.9 |
| Anti-conservative ideation | 18 | 22.8 |
| Suicide attempt | 4 | 5.1 |
| **Non Suicidal Self-injury** | 20 | 25.3 |
| **Obsessive-compulsive disorder** | 16 | 20.3 |
| **Intermittent psychotic symptoms** | 6 | 7.6 |
| **Disperceptive symptoms** | 12 | 15.2 |
| **Nutrition and eating disorders** | 2 | 2.5 |
| **Internet addiction** | 30 | 38.0 |

**Table 2 - suppl**

*Chi-square analysis and distribution of psychiatric comorbidities by cluster*

| **PD/Clusters** | **Psychiatric Comorbidity** | **Chi-Square** | **Cramer's V** | **p-value** |
| --- | --- | --- | --- | --- |
| Cluster A | Social Anxiety | 6.954 | 0.297 | 0.008 |
|  | Externalizing symptoms | 8.743 | 0.333 | 0.003 |
| Schizoid PD | Social Anxiety | 6.550 | 0.288 | 0.010 |
| Schizotypal PD | Psychotic Symptoms | 4.815 | 0.247 | 0.028 |
| Schizoid PD | Externalizing Symptoms | 7.354 | 0.305 | 0.007 |
| Cluster B | Bipolar Spectrum Disorder | 6.679 | 0.291 | 0.010 |
|  | Self-harming Behaviors | 6.434 | 0.285 | 0.011 |
|  | Suicidality | 6.526 | 0.287 | 0.038 |
| Borderline PD | Suicidality | 14.203 | 0.424 | <0.001 |
|  | Bipolar Spectrum Disorder | 8.948 | 0.337 | 0.003 |
|  | Self-harming Behaviors | 21.711 | 0.524 | <0.001 |
| Cluster C | Generalized Anxiety Disorder | 7.264 | 0.303 | 0.007 |
|  | Social Anxiety | 7.264 | 0.303 | 0.007 |
|  | Multiple Anxiety Disorder | 4.702 | 0.244 | 0.030 |
|  | Performance Fear | 8.573 | 0.329 | 0.003 |
|  | School Phobia | 4.470 | 0.238 | 0.034 |
|  | Somatic Symptoms | 7.006 | 0.298 | 0.008 |
| Avoidant PD | Social Anxiety | 5.634 | 0.267 | 0.018 |
|  | Generalized Anxiety Disorder | 5.634 | 0.267 | 0.018 |
|  | Performance Fear | 6.058 | 0.277 | 0.014 |
|  | Somatic Symptoms | 6.167 | 0.279 | 0.013 |

*Note.* Only significant associations are reported, divided by cluster

**Table 3 - suppl**

| *Demographics, cognitive and ADOS-CS comparison between subjects with PD and without PD* | | | | |  |
| --- | --- | --- | --- | --- | --- |
|  | **Type of test** | **Stats** | **gdl** | **p** |  |
| Age | U di Mann-Whitney | 602 |  | 0.241 | |
| Age (months) | U di Mann-Whitney | 602 |  | 0.253 | |
| Tot IQ | Student t-test | 0.1146 | 75.0 | 0.909 | |
| VCI | Student t-test | 0.4127 | 69.0 | 0.681 | |
| PRI | Student t-test | 0.0513 | 73.0 | 0.959 | |
| WMI | Student t-test | -0.1789 | 60.0 | 0.859 | |
| PSI | U di Mann-Whitney | 541 |  | 0.582 | |
| CS-ADOS | Student t-test | -0.4609 | 75.0 | 0.646 | |
| Note. Hₐ μ_0_ ≠ μ_1_  When the data were not normally distributed Mann-Whitney test was used | | | | |  |

**Table 4 - suppl**

*Comparison in CGI, CGAS and questionnaires scores, between subjects with or without PD*

| **Clinical Questionnaire or Clinical Index** | **Subjects without PD** | | **Subjects with PD** | | **Statistical measures** | | |
| --- | --- | --- | --- | --- | --- | --- | --- |
|  | **Mean** | **SD** | **Mean** | **SD** | **p-value** | **Effect size** | **Corrected p-value†** |
| CGAS | 49.64 | 7.641 | 43.65 | 8.806 | 0.003* | 0.712 | 0.037* |
| CGI ° | 4.04 | 1.036 | 4.92 | .913 | <0.001* | -0.450 | 0.019* |
| CBCL_ int_Md | 66.93 | 9.847 | 72.61 | 7.884 | 0.007* | -0.658 | 0.049* |
| CBCL_ ext_Md | 55.57 | 8.430 | 60.57 | 9.933 | 0.028* | -0.531 | 0.087 |
| CBCL_ tot_Md | 62.96 | 9.327 | 68.02 | 7.316 | 0.010* | -0.624 | 0.062 |
| CBCL_1_Anxious/Depressed | 67.11 | 11.087 | 71.63 | 11.211 | 0.091 | -0.405 | 0.161 |
| CBCL_2_Withdrawn depressed | 69.14 | 13.006 | 75.49 | 12.356 | 0.037* | -0.504 | 0.109 |
| CBCL_3_Somatic Compl. ° | 61.93 | 10.026 | 66.61 | 8.897 | 0.060 | -0.259 | 0.131 |
| CBCL_4_Social Problmes ° | 63.43 | 9.211 | 66.49 | 7.754 | 0.063 | -0.255 | 0.131 |
| CBCL_5_Thought problems | 63.89 | 9.975 | 68.71 | 7.961 | 0.023* | -0.552 | 0.082 |
| CBCL_6_Attention problems ° | 61.89 | 9.647 | 63.65 | 9.315 | 0.367 | -0.125 | 0.467 |
| CBCL_7_Rule Breaking behav ° | 55.79 | 5.216 | 58.69 | 6.953 | 0.080 | -0.241 | 0.154 |
| CBCL_8_Aggressive behav ° | 57.50 | 8.289 | 61.90 | 9.040 | 0.021* | -0.319 | 0.082 |
| CBCL DSM Affective problems | 67.89 | 10.254 | 74.51 | 9.033 | 0.004* | -0.697 | 0.037* |
| CBCL DSM Anxiety problems | 67.25 | 7.372 | 69.00 | 8.196 | 0.353 | -0.221 | 0.467 |
| CBCL DSM Somatic problems ° | 59.14 | 9.770 | 63.18 | 9.897 | 0.062 | -0.255 | 0.131 |
| CBCL DSM ADHD ° | 59.50 | 8.452 | 60.35 | 6.854 | 0.480 | -0.098 | 0.56 |
| CBCL DSM ODP ° | 57.50 | 7.886 | 61.39 | 8.366 | 0.040* | -0.282 | 0.112 |
| CBCL DSM Conduct disorder° | 55.39 | 6.112 | 58.59 | 7.724 | 0.092 | -0.231 | 0.161 |
| YSR_ int_Md | 65.32 | 10.931 | 72.15 | 9.725 | 0.007* | -0.670 | 0.049* |
| YSR_ est_Md | 53.18 | 8.654 | 58.96 | 10.274 | 0.015* | -0.596 | 0.07 |
| YSR_tot_Md | 62.14 | 12.146 | 68.41 | 10.114 | 0.019* | -0.574 | 0.082 |
| YSR_1_Anxious/Depressed° | 67.11 | 13.082 | 72.00 | 14.562 | 0.118 | -0.218 | 0.2 |
| YSR_2_Withdrawn/Depressed° | 65.68 | 9.100 | 75.39 | 11.893 | < .001* | -0.470 | 0.019* |
| YSR_3_Somatic Compl. | 60.32 | 7.399 | 64.48 | 11.083 | 0.083 | -0.421 | 0.155 |
| YSR_4_Social Problmes | 62.93 | 9.992 | 68.02 | 10.464 | 0.043* | -0.495 | 0.115 |
| YSR_5_Thought problems° | 62.25 | 9.671 | 68.15 | 10.836 | 0.024* | -0.314 | 0.082 |
| YSR_6_Attention problems° | 62.29 | 11.138 | 68.15 | 13.162 | 0.054 | -0.268 | 0.126 |
| YSR_7_Rule Breaking behav° | 52.68 | 3.486 | 58.35 | 8.367 | < .001* | -0.473 | 0.019* |
| YSR_8_Aggressive behav° | 57.68 | 7.175 | 60.48 | 8.869 | 0.191 | -0.182 | 0.297 |
| YSR DSM Affective problems | 65.54 | 11.003 | 73.15 | 10.362 | 0.004* | -0.718 | 0.037* |
| YSR DSM Anxiety problems° | 64.07 | 9.607 | 65.13 | 10.099 | 0.437 | -0.109 | 0.521 |
| YSR DSM Somatic problems° | 57.57 | 6.914 | 64.17 | 12.382 | 0.045* | -0.278 | 0.115 |
| YSR DSM ADHD° | 59.79 | 8.548 | 61.91 | 8.988 | 0.365 | -0.127 | 0.467 |
| YSR DSM ODP° | 57.71 | 6.863 | 60.33 | 8.380 | 0.244 | -0.162 | 0.342 |
| YSR DSM Conduct disorder° | 54.04 | 5.660 | 59.33 | 9.613 | 0.013* | -0.344 | 0.066 |

*Note.* ° Indicates when Mann-Whtiney test was used due to the lack of normal distribution of the variable

*Indicates statistically significant value (p<0.05)

† FDR correction was applied with Benjamini-Hochberg method

**Table 5 - suppl**

*Comparison in CGI, CGAS and questionnaires scores, between subjects with or without PD*

| **Clinical Questionnaire or Clinical Index** | **Subjects without PD** | | **Subjects with PD** | | **Statistical measures** | | |
| --- | --- | --- | --- | --- | --- | --- | --- |
|  | **Mean** | **SD** | **Mean** | **SD** | **p-value** | **Effect size** | **Corrected p-value †** |
| RIPoSt–Y total | 86.89 | 31.700 | 104.31 | 37.033 | 0.051 | -0.292 | 0.124 |
| RIPoSt–Y Affective instability | 42.74 | 17.233 | 54.17 | 20.989 | 0.025* | -0.588 | 0.082 |
| RIPoSt–Y Emotional reactivity° | 20.30 | 8.398 | 22.43 | 10.021 | 0.430 | -0.119 | 0.521 |
| RIPoSt–Y Interpersonal sensitivity | 23.85 | 9.445 | 27.71 | 9.742 | 0.122 | -0.402 | 0.201 |
| MASC-2 SELF-R tot.° | 62.42 | 11.895 | 63.46 | 15.298 | 0.835 | -0.036 | 0.882 |
| Separation anxiety | 65.89 | 14.854 | 58.32 | 14.399 | 0.073 | 0.296 | 0.146 |
| GAD index | 62.53 | 12.043 | 65.86 | 14.497 | 0.228 | -0.199 | 0.336 |
| Social anxiety total | 63.63 | 11.383 | 63.30 | 13.914 | 0.883 | -0.026 | 0.899 |
| Humiliation/Rejection° | 59.84 | 12.655 | 62.11 | 12.461 | 0.555 | -0.098 | 0.622 |
| Performance fears° | 66.42 | 10.134 | 60.35 | 13.206 | 0.212 | 0.206 | 0.321 |
| Obsessions and compulsions° | 58.37 | 12.415 | 57.41 | 13.961 | 0.550 | 0.100 | 0.622 |
| Physical symptoms tot° | 60.26 | 12.662 | 63.62 | 15.578 | 0.377 | -0.147 | 0.469 |
| Panic ° | 57.21 | 13.439 | 61.35 | 16.471 | 0.303 | -0.171 | 0.414 |
| Tense/Restless° | 58.68 | 11.289 | 62.22 | 14.060 | 0.972 | 0.007 | 0.972 |
| Harm Avoidance° | 50.00 | 8.660 | 50.73 | 10.527 | 0.883 | -0.026 | 0.899 |
| ICU tot° | 24.24 | 7.645 | 30.17 | 13.155 | 0.127 | -0.232 | 0.203 |
| ICU callousness° | 7.72 | 4.228 | 11.78 | 6.508 | 0.012* | -0.379 | 0.066 |
| ICU anemotionality° | 7.68 | 3.388 | 8.86 | 4.128 | 0.243 | -0.307 | 0.342 |
| ICU indifference | 9.08 | 3.581 | 9.75 | 5.505 | 0.595 | -0.139 | 0.653 |
| SCQ-life span | 10.88 | 5.567 | 10.47 | 5.643 | 0.763 | 0.074 | 0.822 |

*Note.* ° Indicates when Mann-Whitney test was used due to the lack of normal distribution of the variable

*Indicates statistically significant value (p<0.05)

† FDR correction was applied with the Benjamini-Hochberg method

**Supplementary Material – SCID-5-PD Adaptations for Adolescent Assessment**

The SCID-5-PD interview was administered in Italian by trained clinicians with expertise in developmental psychopathology. Minor linguistic and contextual adaptations were applied to ensure developmental appropriateness while preserving the original meaning and diagnostic intent of each item.

**Linguistic adaptations**

Selected questions were rephrased into developmentally accessible language, avoiding overly abstract or adult-oriented formulations. When necessary, clinicians provided brief clarifications or examples to facilitate comprehension, while maintaining fidelity to the underlying construct assessed. For example, items referring to “interpersonal relationships” or “identity” were framed using age-appropriate terms such as friendships, school interactions, family dynamics, and self-image.

**Contextual adaptations**

Questions were anchored to contexts that are salient during adolescence, including school environment, peer relationships, family interactions, and online/social media experiences. This approach allowed clinicians to evaluate the presence and pervasiveness of personality-related patterns across relevant developmental settings.

**Developmentally informed interpretation**

Given the ongoing maturation of personality during adolescence, particular care was taken to distinguish enduring and pervasive maladaptive patterns from transient, developmentally normative behaviors. Traits were considered clinically meaningful only when they were stable over time, present across contexts, and associated with significant impairment.

**Cautious interpretation in the context of autism**

Special attention was paid to the potential overlap between autism-related characteristics and personality disorder criteria. When evaluating SCID-5-PD items, clinicians considered whether observed behaviors (e.g., social withdrawal, rigidity, emotional dysregulation) could be better accounted for by core autism features, sensory distress, or adaptive responses to environmental stressors (e.g., camouflaging, social exclusion), rather than reflecting distinct personality pathology.
